# Do People Believe They Are Less Predictable Than Others? Three Replications of Pronin and Kugler’s (2010) Experiment 1

**DOI:** 10.5334/irsp.946

**Published:** 2024-12-06

**Authors:** Subramanya Prasad Chandrashekar, Stephanie Permut, Hallgeir Sjåstad, Chelsea (Chi Wing) Lo, Yong Jun Kueh, Emily Sihui Zhong, Kai Hin Wan, Kai Yi Kelly Choy, Man Chung Wong, Stanley Wei Jian Hugh, Khan Tahira, Bo Ley Cheng, Gilad Feldman

**Affiliations:** 1 Department of Psychology, Norwegian University of Science and Technology (NTNU), NO; 2 Andlinger Center for Energy and the Environment Princeton University, US; 3 Department of Strategy and Management, Norwegian School of Economics, NO; 4 Department of Psychology, University of Hong Kong, HK

**Keywords:** predictability, self-other asymmetry, free will attributions, replication

## Abstract

Pronin and Kugler (2010) proposed that people believe they have more free will than others. In their Experiment 1 they showed that US students evaluated their own decisions and life events as less predictable than similar decisions and life events of close others, presumably suggesting higher free will attributions. We conducted three pre-registered replications of this study, one with a Hong Kong undergraduate sample (*N* = 47) and two online samples from the USA (MTurk using CloudResearch: *N* = 126, Prolific: *N* = 858) (overall *N* = 1031). In Studies 1a and 1b that mirrored the target article’s mixed design (self-other between, past-future within), we found support for the original findings with weaker effects. In Study 2 we contrasted between-subject versus within-subject designs in a single data collection. We successfully replicated the effects with the between-subject design, whereas we failed to find support for the effect using the within-subjects design. This suggests support for the phenomenon in single evaluation mode assessing either the self or the other, but that people correct for the self-other asymmetry in perceived predictability when the judgment is made in joint evaluations mode. Materials, data, and code are available on: https://osf.io/ykmqp/. Open peer review: https://osf.io/d47kj.

Do people believe that they have more free will than others? An influential study by Pronin and Kugler (2010) provided initial evidence for this hypothesis, in which their Experiment 1 showed that people believe that their own decisions and life outcomes are less predictable than similar decisions and life outcomes of others. The link the authors made to free will, was that higher predictability of past and future events would presumably be at odds with personal agency, at least in laypersons’ views, thereby leaving less room for freedom of choice.

In the current research, we report three pre-registered replications of this experiment using different samples and variations of study designs to reproduce, replicate, and test the generalizability of this effect.

## ‘Free Will’ Research in Psychology

Free will is often associated with the capacity for making choices between alternatives without internal or external constraints (Feldman et al. 2014; Monroe et al. 2014; Rigoni et al. 2017). Most laypersons seem to believe in some form of free will (Alquist et al. 2015; Monroe & Malle 2010; Nahmias et al. 2005; Nichols 2004; Sarkissian et al. 2010; Stillman et al. 2011). In philosophy, there is extensive debate about the very existence of free will, how it should be conceptualized in formal terms, whether it is compatible with determinism and biological evolution, and what the implications of free will might be for ethics and society (e.g., Dennett & Caruso 2021). In social psychology and experimental philosophy, the focus has shifted towards using empirical research methods to study how ordinary people think and feel about free will, and to examine the causes, consequences, and correlates of people’s free will beliefs and attributions. The study by Pronin and Kugler has been one of the first to suggest interesting asymmetries in the way that people tend to think about concepts related to free will when reflecting on their own compared to that of others’ life circumstances.

People readily make inferences about the degree of personal agency involved in a decision, both when reflecting on their own behavior and on the behaviors of others (Gray et al. 2012). Previous research documented associations between free will beliefs with motivation and work performance (Stillman et al. 2010), psychological well-being, and adjustment (Crescioni et al. 2016), stricter moral judgments of blame and punishment (Martin et al. 2017; Shariff et al. 2014). It therefore seems that free will beliefs are associated with a broad range of factors that might impact people’s lives. We therefore sought to conduct an independent replication of what we considered to be one of the first central findings in this literature, suggesting that people believe that they have more free will than other people by being less predictable a priori.

Early replication attempts in free will research have focused on the replicability of previous findings related to the consequences of free will beliefs. The well-known original studies on these consequences by using a short and simple belief activation technique (e.g., Alquist et al. 2015; Clark et al. 2014; Vohs & Schooler 2008). Recent replication efforts evaluating free will belief activation tasks have mostly failed to support the original findings (Buttrick et al. 2020; Embley et al. 2015; Genschow et al. 2021; Katzir & Genschow 2022; Nadelhoffer et al. 2020) with a recent meta-analysis of the literature concluding that despite a successful manipulation check there is no evidence for any downstream consequences (Genschow et al. 2023). These failed replications mirror broader concerns in psychology around social priming studies which claim to use similar activation techniques (Chivers 2019).

A more promising approach seems to be correlational research on associations with individual differences in free will beliefs (e.g., Martin et al. 2017), or experiments regarding free will attributions that vary one aspect of a hypothetical choice situation before eliciting agency attributions and related judgments (e.g., Everett et al. 2021; Feldman et al. 2016, 2018). For instance, recent work by Fillon et al. (2022) found that people attributed higher free will to exceptional behavior compared to routine behavior, particularly when the exceptional actions were a result of personal choice rather than external factors. Similarly, Genschow and Lange (2022) found that belief in free will relates to higher internal attributions for one’s own actions.

Pronin and Kugler (2010) used a variant of the latter method and randomly assigned participants to evaluate free will related indicators regarding their own lives or the lives of others. In the case of Experiment 1, they tested people’s attributions of *predictability* regarding past and future life events, contrasting evaluations of one’s own life compared to that of other people.

## Self-Other Asymmetries in Human Judgement

There are many documented phenomena of self-other asymmetries. One of those is ‘unrealistic optimism,’ which is the phenomenon that people tend to believe they are more likely to experience positive events and life outcomes than is warranted, and to underestimate their personal risks (Shepperd et al. 2015; Weinstein 1980). Research on the ‘better-than-average effect’ has documented that people have a broad tendency to view themselves in a favorable light when comparing themselves to others (Alicke 1985; Svenson 1981; see successful replication by: Koppel et al. 2023; Ziano et al. 2021), though over time identifying some nuances and moderators, such as an interaction with difficulty (Kruger 1999).

Pronin and Kugler (2010) found that people attribute more free will to themselves both for positive and negative outcomes, which suggested a distinct phenomenon from the classic form of optimism and better-than-average effects. Drawing on the ‘actor-observer bias’ (Jones & Nisbett 1971), Pronin and Kugler (2010) proposed that people might perceive the actions of others as fixed dispositions of their stable and uncontrollable personalities, whereas people perceive their own actions as intentional unconstrained responses to changing situations. This latter perspective presumably leaves more room for free will to operate. The authors did not explicitly explain how unpredictability relates to the philosophical standpoint of indeterminism—that not all events, including human actions, are determined by preceding events or natural laws. Our understanding of their point is that if actions are unpredictable, then it suggests that they are not wholly dictated by prior states of the world, thus allowing for genuine choice or volition (Kane 1996). We note that there is at least one other possibility in that unpredictability is unrelated to determinism and has more to do with human’s ability to predict, whether determined or not. It remains unclear what the link is between unpredictability and free will, and whether laypeople associate unpredictability with free will. We return to this point in our discussion.

## Cognitive Neglect Versus Self-Enhancement Motives

The self-other asymmetry observed in free will attributions may stem from the self-enhancement motive, wherein individuals tend to perceive themselves more favorably than the average person across various domains, including intelligence, skill, and positive personality traits (Alicke 1985; Alicke et al. 1995; Ziano et al. 2021). Alternatively, this asymmetry could be partially or entirely influenced by a cognitive bias, where individuals rely on introspective information, such as feelings and intentions, for self-assessments. Considering these possibilities, it can be argued that if the self-other asymmetry is primarily driven by self-enhancement motives, it will persist in within-person comparisons. Conversely, the absence of this asymmetry in within-person comparisons suggests that cognitive neglect, which individuals can potentially correct for, plays a significant role in driving the asymmetry. We proposed an extension study to evaluate this possibility.

## Extension: Examining Bias, by Contrasting Within and Between Subject Designs

It remains an open question what research participants themselves would make of their tendency to claim being less predictable than others. As that experiment was based on a between-subjects self-other design, where participants either rated the level of predictability in their own lives *or* in the lives of close others, the systematic inconsistency between conditions remain ‘invisible’ to individuals making those ratings. Thus, in addition to examining the replicability of Pronin and Kugler’s (2010) main finding, in our Study 2 we extended their original design with a variety of contrasts (fully within-subject conditions, a fully between-subject condition, and mixed-design) within a single data collection.

Previous studies suggested that if a given effect is driven by cognitive neglect or irrational bias, then the effect will tend to be weaker or disappear when tested in a within-subjects design. This is because the within-subjects structure facilitates direct comparison in joint evaluation (Caruso et al. 2008; Hsee et al. 1999; Kahneman & Ritov 1994). Based on this logic, we reasoned that if we observe that people in a within-subjects condition do not show the effect, then we might interpret this as cognitive neglect, and less likely to have a motivational origin. That is, people can recognize the inconsistency when it is made clear by contrasting conditions against one another in the same experiment (for a recent example of this method, see Sjåstad et al. 2020).

## Studies Overview

In the current research, we report three pre-registered replications of Experiment 1 in Pronin and Kugler (2010). For the target study, they observed very large effects. Our Studies 1a and 1b were close replications of the original study design, with initial small samples, which we considered to be pre-tests. In Study 2, we followed up with a much larger sample, also aiming to examine the possible role of cognitive neglect versus motivational self-enhancement as mechanisms. In Study 2 we included an extension contrasting the fully within-subject condition with the fully between-subject condition, adjusted from the original mixed design. Study 2 also included a trait achievement measure as a possible individual-difference moderator of the effect. We summarized the replication hypotheses in Table 1 and provided an overview of the findings of Experiment 1 of the target article in Table S17.

**Table 1:** Pronin and Kugler (2010): Summary of hypotheses.


STUDY	HYPOTHESIS

Hypothesis 1	People perceive their own past and future decision as less predictable a priori than those of a roommate.

Hypothesis 2a	People perceive their own past decisions as less predictable than the ones of others.

Hypothesis 2b	People perceive their own future decisions as less predictable than the ones of others.

Hypothesis 3	People perceive future decisions as less predictable than past decisions.


### Open Science, Preregistrations, and Disclosures

Materials, data, and code are available on the Open Science Framework (project: https://osf.io/ykmqp/; preregistration Study 1a: https://osf.io/jg94e; preregistration Study 1b: https://osf.io/yaepf/; preregistration Study 2: https://osf.io/sjb8p). All studies, participants, measures, manipulations, and exclusions conducted for this investigation are reported, and data collection was completed before hypothesis testing.

### Planned Sample

We calculated effects in Pronin and Kugler (2010) using available F-statistics and sample sizes for each condition with the help of a guide by Jané et al. (2024). The effect size (Cohen’s *d*) of 1.1 for the between-subject factor (self vs. other) was used as the basis of power analysis.

In Studies 1a and 1b, we based our power analysis on having 95% (*α* = 0.05) power to detect a Cohen’s *d* = 1.08. This resulted in a suggested sample size of 46 across two between-subject conditions. More detailed descriptions of the effect-size calculations and power analyses can be found in the supplementary materials.

In Study 2, we determined the number of participants needed to detect an effect size of *d* = 0.32 at 95% power (*α* = 0.05). The effect size of *d* = 0.32 was based on the results of Study 1b. Our analysis yielded a sample of 426 participants for studies involving between-subjects effects and a sample of 108 participants for studies involving within-subject effects. To ensure that both designs were conducted with adequate statistical power, aiming for a conservative estimate, we took the largest sample size needed to detect a moderate between-subjects effect and set this as our target sample.

### Deviations

We provided detailed information of designs (type of study, sample, variables, exact wordings) of the original studies in the Methods and Original articles’ results section of the Supplementary materials. We noted several deviations from the original, summarized in Table S15 and Table S16.

### Replications Classification

We categorized Studies 1a and 1b as ‘very close replications’ and Study 2 as a ‘close replication’ based on the criteria by LeBel et al. (2018), with our classification analysis provided in Table S16.

## Study 1a: Hong Kong Student sample

Study 1a was our first attempt to replicate the effect, conducted on an undergraduate student sample in Hong Kong. The experiment was run together with a series of other judgment and decision-making studies that were designed by the students in the sample and ran together in randomized order on the designing students. Therefore, given that students were familiar with other judgment and decision-making phenomena and the relatively small sample, we consider this study to be a conservative pre-test replication on a well-informed sample.

### Method

#### Participants

We recruited a small sample of 47 participants (*M_age_* = 20.2, *SD_age_* = 1.02; 16 males, 31 females). A sensitivity analysis indicates that this sample can detect effects of *d* = 0.97 (one-tail, 95% power), an effect smaller than that in the target article, yet we considered this a pre-test. Materials were presented in an online survey using the Qualtrics software. We collected no demographic information in this study.

#### Procedure

Participants were randomly assigned to one of two conditions: ‘self’ and ‘other’. Participants in the ‘self’ condition judged the predictability of past and future events in their own lives whereas participants in the ‘other’ condition judged the predictability of past and future events in the life of a close other. We prompted participants in the ‘other’ condition to provide the initials of the person they had in mind before proceeding to provide their predictability ratings.

We ran a 2 target (self vs. other; between-subjects) × 2 time (past vs. future; within-subjects) mixed study design. Participants evaluated three target past events (1 = *Not at all predictable*, 7 = *Extremely predictable*): 1) chosen university for studies, 2) chosen major for studies, and 3) failure in dating, and three future events: 1) career choice, 2) person to marry, 3) place to live, six target events in total.

#### Exclusions

We pre-registered an exclusion criteria (failing to complete the entire survey, failing to adhere to pre-test requirements, indicating low English proficiency, and reporting low seriousness), yet no participants were excluded as a result of these exclusion criteria. The students who designed the study also participated but their answers were excluded from the analysis.

### Results

**Figure 1:**
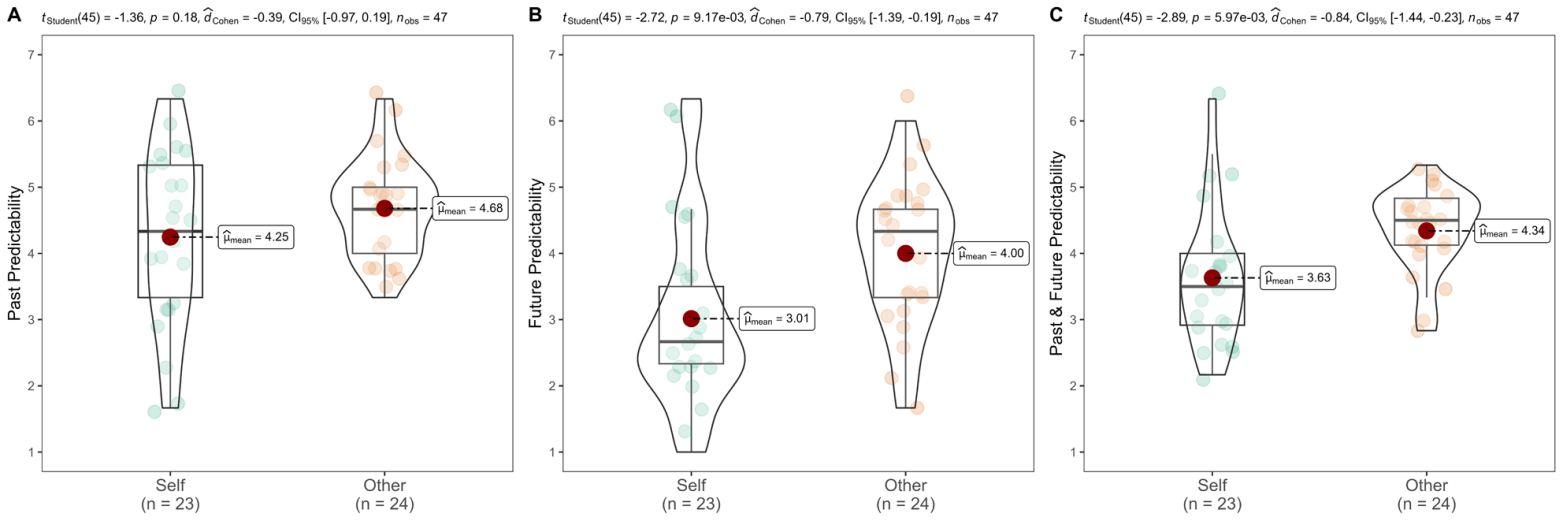
Study 1a: Predictability – self vs. other.

We summarized descriptives in Table 2 and Figure 1.

**Table 2:** Study 1: Predictability descriptives.


TARGET [BETWEEN]	SELF (*N* = 23)		OTHER (*N* = 24)	
	
TIME [WITHIN]	*M*	*SD*	*M*	*SD*

Past	4.25	1.39	4.68	0.72

Future	3.01	1.39	4.00	1.08

Overall	3.63	1.02	4.34	1.02


We conducted an independent t-test and found support for the findings in Pronin and Kugler (2010). Participants viewed their own decisions as being less predictable than the decisions of close others (self: *n* = 23, *M* = 3.63, *SD* = 1.02; other: *n* = 24, *M* = 4.34, *SD* = 0.62; *t* (45) = –2.89, *p* = .003, one-tailed, *d* = –0.84, 95% CI [–1.46, –0.23]).

Examining past and future separately, future events for self (*M* = 3.01, *SD* = 1.39) were rated as less predictable than others’ (*M* = 4.00, *SD* = 1.08; *t* (45) = –2.72 *p* = .005, one-tailed, *d* = –0.79, 95% CI [–1.41, –0.18]). We did not find support for past events, as past events related to self (*M* = 4.25, *SD* = 1.39) were rated similarly to others’ past events (*M* = 4.68, *SD* = 0.72; *t* (45) = –1.36, *p* = .091, one-tailed, *d* = –0.40, 95% CI [–0.99, 0.20]). Therefore, the effects were stronger for the future than for the past, although given the small sample we did not detect support for interaction. The results of the mixed ANOVA analysis are reported in the supplementary section (see Table S3 and S4).

Consistent with the original findings, there also was a main effect for time, whereby people perceived the future as less predictable than the past (*M* = 3.52 (*SD* = 1.32) versus 4.47 (1.11); *t* (46) = –3.99, *p* < .001, one-tailed, *d_rm_* = –0.78, 95% CI [–1.22, –0.34], *r* = 0.11).

## Study 1b: US Americans on MTurk

### Method

#### Procedure and Exclusions

We recruited an online sample of 136 US students from Amazon Mechanical Turk using CloudResearch/TurkPrime (Litman et al. 2017). All study materials and instructions were identical in structure and content to the survey used in Study 1a, with a 2 (target: self vs. other; between-subjects) by 2 (time: past vs. future; within-subjects) mixed study design.

Four participants failed to give consent to partake in the study, and four other participants indicated that they were not currently American students, and therefore were not allowed to advance past the consent form of the survey. Two additional participants did not complete the study. Our final sample consisted of 126 participants (*M_age_* = 25.83, *SD_age_* = 6.46; 63 female, 63 male).

### Results and Discussion

**Figure 2:**
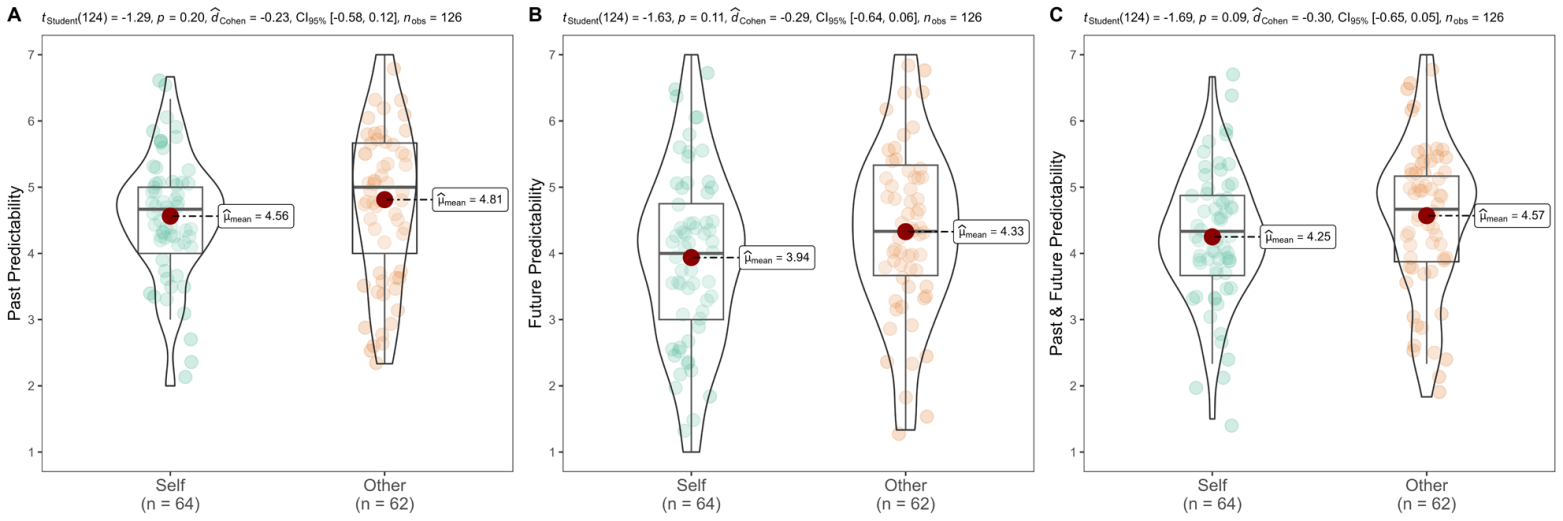
Study 1b: Self vs. other predictability.

We summarized descriptives in Table 3 and Figure 2.

**Table 3:** Study 1b: Predictability descriptives.


TARGET [BETWEEN]	SELF (*N* = 64)		OTHER (*N* = 62)	
	
TIME [WITHIN]	*M*	*SD*	*M*	*SD*

Past	4.56	0.92	4.81	1.22

Future	3.94	1.36	4.33	1.34

Overall	4.24	0.95	4.58	1.15


We conducted independent *t*-tests and the effects were in the expected direction, yet we failed to meet the evidence criteria we set for concluding support for the findings in Pronin and Kugler (2010). Participants viewed their own decisions as being less predictable than the decisions of close others (self: *n* = 64, *M* = 4.25, *SD* = 0.95; other: *n* = 62, *M* = 4.57, *SD* = 1.16; *t*(124) = –1.69, *p* = .046, one-tailed, *d* = –0.30, 95% CI [–0.66, 0.05]).

Examining past and future separately, future events for self (*M* = 3.94, *SD* = 1.36) were rated as less predictable than others’ (*M* = 4.33, *SD* = 1.34; *t*(124) = –1.63, *p* = .053, one-tailed, *d* = –0.29, 95% CI [–0.64, 0.06]). We did not find support for past events, as past events related to the self (*M* = 4.56, *SD* = 0.92) were rated similarly to others’ past events (*M* = 4.81, *SD* = 1.22; *t*(124) = –1.29, *p* = .099, one-tailed, *d* = –0.23, 95% CI [–0.58, 0.12]). Therefore, the effects were stronger for the future than for the past, although given the small sample we did not detect support for interaction. Additionally, we found support for the main effect of time, whereby people perceived the future as less predictable than the past (*M* = 4.69 (*SD* = 1.08) vs. 4.13 (1.36); *t*(125) = –5.14, *p* < 0.001, one-tailed, *d_rm_* = –0.45, 95% CI [–0.63, –0.27], *r* = 0.53). The results of the mixed ANOVA analysis are reported in the supplementary section (see Table S5 and S6).

The results of Studies 1a and 1b indicated some support for its findings, across two samples from the USA and Hong Kong. The original effect estimates were in the predicted direction, yet we caution against overinterpretation given the weak effects and just-below threshold one-tail p-values. Especially so given that we were not clear enough in advance in our pre-registrations about whether we would employ one or two-tail analyses, only that we conducted our power analyses using one-tail as the base for calculations. Therefore, we provide our data as is. We followed our pre-registered criteria, and tried to avoid any flexibility in analysis, yet recognize the challenge with one-tail p-values just below .05. The reporting of one-tail seems reasonable given that this was a replication with clear directional hypotheses and our power analyses.

However, we felt that given the weak effects and just-below-threshold one-tailed *p*-values, caution is warranted, and therefore proceeded to conduct another well-powered data collection, with added extensions.

## Study 2: US Americans on Prolific

We considered Study 2 as a well-power repeat of the study including extensions testing different study designs.

Participants were randomly assigned to one of the following experimental design conditions:^2^ 1) a fully between-subjects design, 2) a fully within-subject design, and 3) a mixed design. All study materials and instructions were identical in structure and content to the survey used in previous studies. We focused our analyses below on the first two conditions, given an oversight in the third condition that we felt limited its contribution, and its analyses are reported in the supplementary materials.

The study participants in the fully between-subjects design were randomly assigned to one of four conditions in a between-subjects design: 2 (Target: self vs. other) × 2 (Time: past vs. future). Whereas the participants in the fully within-subjects design answer all four combinations of Target (self, other) and Time (past, future).^1^

### Method

#### Participants

We recruited a total of 1304 online student participants using Prolific participant recruitment platform. We pre-registered several exclusion criteria for this study: Low English proficiency, reporting not being serious, or if they failed to complete the study. In total, we excluded 9 participants, which resulted in a sample of 1295 that met our exclusion criteria (*M_age_* = 20.4, *SD_age_* = 1.93, 525 males, 770 females).

#### Procedure

After responding to the consent form, participants completed a 10-item version of the Achievement Motives Scale (Lang & Fries 2006). They were then randomly assigned to view one of three study designs: fully between-subjects (*n* = 424), fully within-subjects (*n* = 434) or the mixed condition (*n* = 437). We note that we focused our analysis on the fully between and fully within design conditions, and the analysis of the mixed-subject condition has been moved to the supplementary materials.^2^ Materials and measures were the same as those used in Studies 1a/1b.

### Results

#### Between-Subject Design

**Figure 3:**
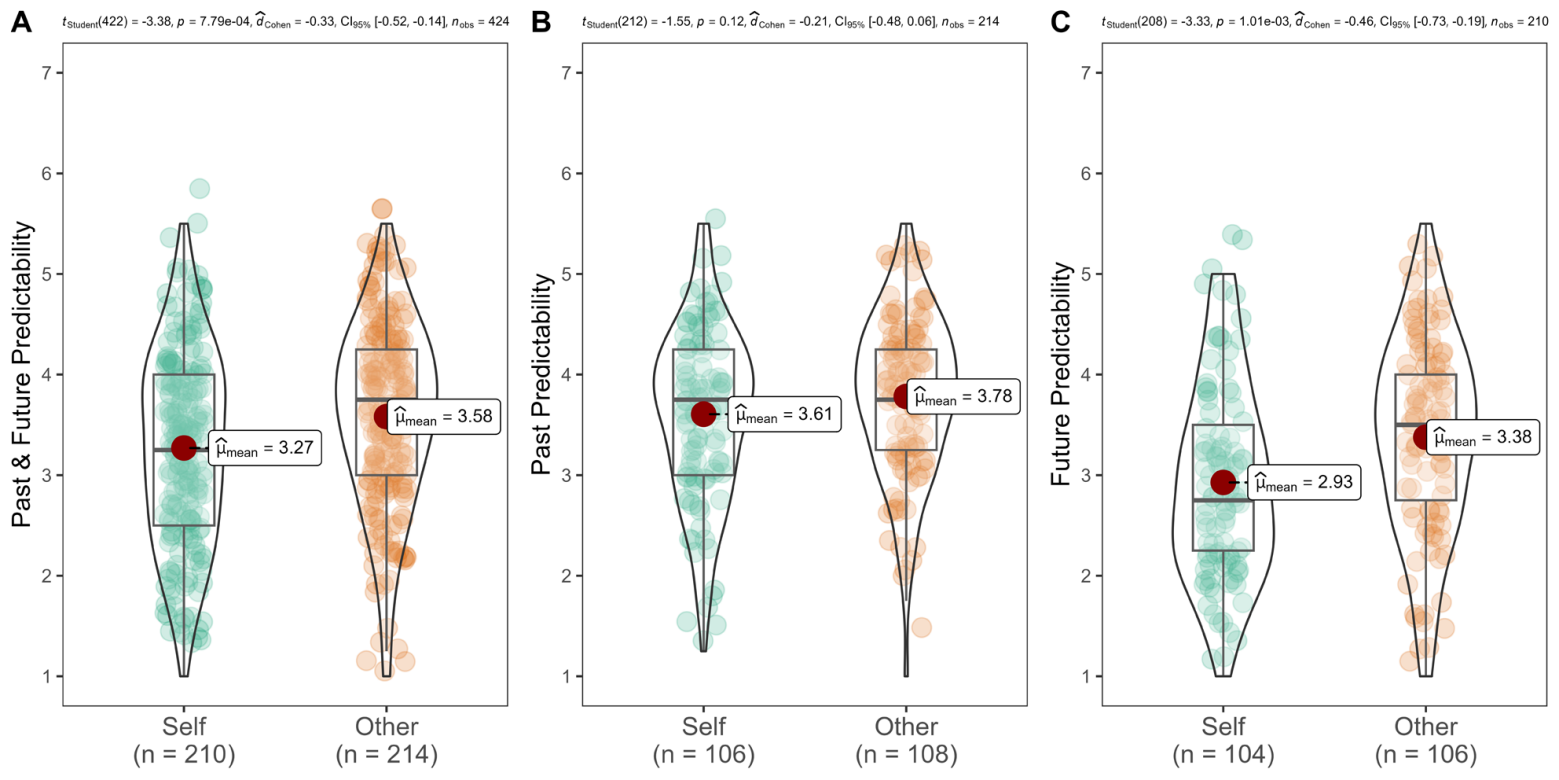
Study 2 between-subjects design: self vs. other predictability.

We summarized descriptives in Table 4 and Figure 3.

**Table 4:** Study 2 between design: Predictability descriptives.


TARGET [BETWEEN]	SELF			OTHER		
	
TIME [BETWEEN]	*N*	*M*	*SD*	*N*	*M*	*SD*

Past	106	3.61	0.86	108	3.78	0.81

Future	104	2.93	0.97	106	3.38	0.99

Overall	210	3.27	0.97	214	3.58	0.923


We conducted independent *t*-tests and the effects were in the expected direction. Participants viewed their own decisions as being less predictable than the decisions of close others (self: *n* = 210, *M* = 3.27, *SD* = 0.97; other: *n* = 214, *M* = 3.58, *SD* = 0.92; *t* (422) = –3.38, *p* < .001, one-tailed, *d* = –0.33, 95% CI [–0.52, –0.14]). Examining past and future separately, future events for self (*M* = 2.93, *SD* = 0.96) were rated as less predictable than others’ (*M* = 3.38, *SD* = 0.99; *t* (208) = –3.33, *p* < .001, one-tailed, *d* = –0.46, 95% CI [–0.74, –0.18]). We did not find support for past events, as self past events (*M* = 3.61, *SD* = 0.86) were rated similarly to others’ past events (*M* = 3.78, *SD* = 0.81; *t* (212) = –1.55, *p* = .061, one-tailed, *d* = –0.21, 95% CI [–0.48, 0.06]). Therefore, the effects were stronger for the future than for the past. Additionally, we found support for a main effect for time, whereby people perceived the future as less predictable than the past (*M* = 3.15 (*SD* = 1.00) vs. 3.70 (0.84); *t* (422) = –6.04, *p* < .001, one-tailed, *d* = –0.59, 95% CI [–0.78, –0.39]). The results of the 2-way analysis of variance (ANOVA) analysis are reported in the supplementary section (see Table S7 and S8).

#### Within-Subjects Design

**Figure 4:**
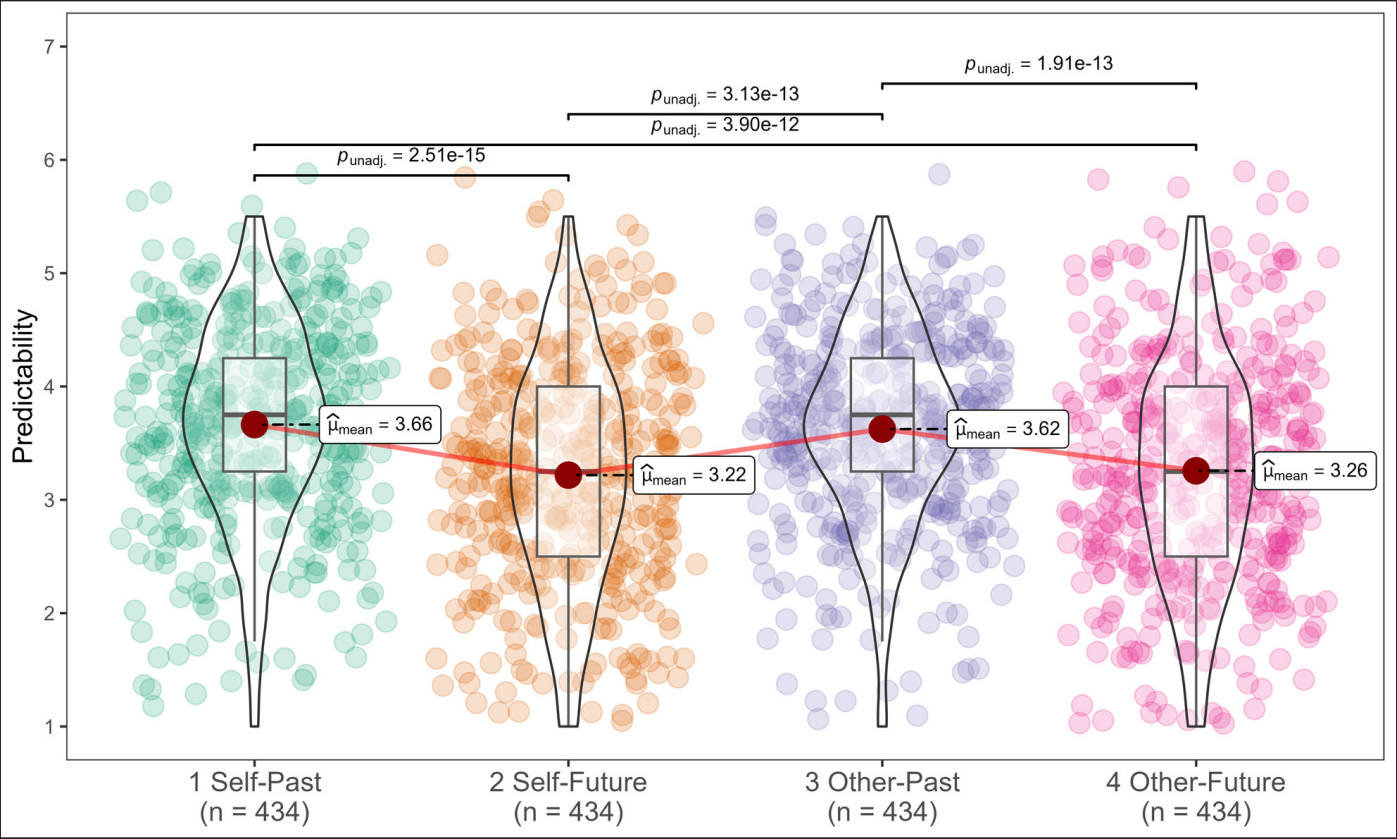
Study 2 within-subjects design: self vs. other predictability.

We summarized the descriptives in Table 5 and Figure 4. We conducted paired *t*-tests as part of the analyses. In joint evaluation mode, we found no support for participants viewing their own decisions as being less predictable than the decisions of close others (self: *M* = 3.44, *SD* = 0.96; other: *M* = 3.44, *SD* = 0.95; *t* (433) = –0.01, *p* = .490, one-tailed, *d_rm_* = 0.00, 95% CI [–0.10, 0.10], *r* = 0.44). Examining past and future separately, we found no support for differences between future events for self (*M* = 3.22, *SD* = 0.99) and for others’ (*M* = 3.26, *SD* = 1.00; t (433) = –0.724, *p* = .230, one-tailed, *d_rm_* = –0.04, 95% CI [–0.14, 0.07], *r* = 0.38). Similarly, we did not find support for past events, as self past events (*M* = 3.66, *SD* = 0.88) were rated similarly to others’ past events (*M* = 3.62, *SD* = 0.85; *t* (433) = 0.821, *p* = .790, one-tailed, *d_rm_* = 0.05, 95% CI [–0.06, 0.15], *r* = 0.33).

**Table 5:** Study 2 within-subjects design: Predictability descriptives.


TARGET [WITHIN]	SELF		OTHER	
	
TIME [WITHIN]	*M*	*SD*	*M*	*SD*

Past	3.66	0.89	3.62	0.85

Future	3.22	0.99	3.26	1.00

Overall	3.44	0.96	3.44	0.95


*Note*. n = 434.

However, we found support for the effect of time, people perceived the future (*M* = 3.24, *SD* = 0.99) as less predictable than the past (*M* = 3.64, *SD* = 0.87; *t* (433) = –10.30, *p* < .001, one-tailed, *d_rm_* = –0.53, 95% CI [–0.64, –0.42], *r* = 0.43). We summarized the results of the within-subjects ANOVA analyses in the supplementary section (see Table S9 and S10).

### Discussion

Study 2 involved a larger sample and modified experimental designs to test the robustness of the findings of the Experiment of Pronin and Kugler (2010). The results based on the between-subjects design supported the predictions of the original study. However, the results based on the novel within-subjects and mixed designs failed to support and extend the original findings, which can shed new light on potential mechanisms. Additionally, there was no support for the moderation effect of achievement motivation as individual-level factor.

## Comparing Replication Findings to Original Findings and Extensions

We compiled a comparison between the effects of the original studies and our replications in Table 6, along with an interpretation of the findings using the evaluation criteria established by LeBel et al. (2019) for replication results.

**Table 6:** Replication results compared to original effects.


SAMPLE	PREDICTOR	REPLICATION RESULTS	ORIGINAL STUDY	REPLICATION	INTERPRETATION

**Study 1a**	H1: General (self-other)	*F*(1,45) = 8.33, *p* = .006	–1.08[–1.67,–0.48]	–0.84[–1.46,–0.23]	Signal-consistent

	H2a: Past outcomes (self-other)	*t*(45) = –1.36, *p* = .181	–0.72[–1.29,–0.14]	–0.40[–0.99,0.20]	No signal-inconsistent

	H2b: Future outcomes (self-other)	*t*(45) = –2.72, *p* = .009	–0.82[–1.40,–0.25]	–0.79[–1.41,–0.18]	Signal-consistent

	H3: Future-past	*F*(1,45) = 16.26, *p* < .001	–0.94[–1.52,–0.35]	–0.77[–1.22,–0.34]	Signal-consistent

	H1: General (self-other)	*F*(1,124) = 2.87, *p* = .093	–1.08[–1.67,–0.48]	–0.30[–0.65,0.03]	No signal-inconsistent

**Study 1b**	H2a: Past (self-other)	*t*(124) = –1.29, *p* = .198	–0.72[–1.29,–0.14]	–0.23[–0.58,0.12]	No signal-inconsistent

	H2b: Future (self-other)	*t*(124) = –1.63, *p* = .106	–0.82[–1.40,–0.25]	–0.29[–0.64,0.06]	Signal-inconsistent

	H3: Future-past	*F*(1,124) = 26.22, *p* < .001	–0.94[–1.52,–0.35]	–0.45[–0.70,–0.20]	Signal-inconsistent(smaller)

**Study 2 (between condition)**	H1: General (self-other)	*F*(1,420) = 12.59, *p* < .001	–1.08[–1.67,–0.48]	–0.33[–0.52,–0.14]	Signal-inconsistent(smaller)

	H2a: Past (self-other)	*t*(420) = –1.42, *p* = .156	–0.72[–1.29,–0.14]	–0.21[–0.48,0.06]	No signal-inconsistent

	H2b: Future (self-other)	*t*(420) = –3.58, *p* < .001	–0.82[–1.40,–0.25]	–0.46[–0.74,–0.18]	Signal-inconsistent(smaller)

	H3: Future-past	*F*(1,420) = 37.74, *p* < .001	–0.94[–1.52,–0.35]	–0.59[–0.78,–0.39]	Signal-inconsistent(smaller)

**Study 2 (within condition)**	H1: General (self-other)	*F*(1,433) = 0.00, *p* = .988	–1.08[–1.67,–0.48]	0.00[–0.10,0.10]	No signal-inconsistent

	H2a: Past (self-other)	*t*(433) = 0.04, *p* = .411	–0.72[–1.29,–0.14]	0.05[–0.06,0.15]	No signal-inconsistent

	H2b: Future (self-other)	*t*(433) = –0.04, *p* = .469	–0.82[–1.40,–0.25]	–0.04[–0.14,0.07]	No signal-inconsistent

	H3: Future vs. past	*F*(1, 433) = 105.23, *p* < .001	–0.94[–1.52,–0.35]	–0.53[–0.64,–0.42]	Signal-inconsistent(smaller)


The target study’s main prediction (*H1*) was that people view their own past and future decisions as less predictable a priori than those of others. Our direct replication samples in Studies 1a and 1b supported the prediction, though with weaker effects and just barely below the threshold in Study 1b. We observed similar patterns for *H2a* and *H2b*, yet slightly stronger support for the hypothesis for the future than for the past. Also, we found evidence supporting the prediction (*H3*) that individuals perceive the future as less predictable than the past.

In Study 2, we juxtaposed two supplementary designs within a single data collection: fully between-subject, and fully within-subject. The main effect (H1) was replicated in the between-subject condition, whereas we found no support for the effect in a new within-subject condition.

We also conducted the mini-meta-analyses with a random-effects model method using the *meta* package in R (Schwarzer, 2007; see *Figure S9–S12* for detailed forest plots in the Supplementary Materials section). The analysis included effects from Study 1a, Study 1b, and Study 2. Among effect sizes from Study 2, we only considered effects from between-subject design condition. The results support the main prediction (*H1*) that people view their own past and future life decisions as less predictable a priori than those of a roommate (*d* = –0.36 [–0.52, –0.20]).

## General Discussion

We aimed to closely replicate Pronin and Kugler’s (2010) Experiment 1, which found that people view their own decisions and life events as less predictable than others’. We also went beyond the original findings by adding extensions examining different study designs.

Across three replications we concluded a mostly successful replication of the self-other asymmetry in predictability ratings, reported in Pronin and Kugler’s (2010) Experiment 1: People rate themselves as being less predictable than others. The effect sizes were considerably smaller than that reported in the original study. We found much weaker to no support for the effect when we ran a novel within-subject design. This suggests that the tendency to believe that oneself is less predictable than other people only emerged in separate evaluation mode, in which the participant makes no direct comparison between herself and others. When the response mode shifts from separate to joint evaluation, people can directly compare their self and other judgments, and then seem to correct this bias in their judgments.

Our replications differed from the original study in some expected ways. For example, stimuli used in the original article were targeted at and tested with US undergraduates more than a decade ago. We used the same materials in an online survey with a more diverse population in Studies 1b and 2. Direct close replications are never perfectly exact, yet despite the differences, we believe that the theoretical grounds of the target paper remain relevant and that the replications indicate a qualified support for the target article’s primary hypothesis.

It might be possible to make the link between the current findings and the phenomenon that people view their own judgments as less biased than others’. For example, Pronin et al. (2002) demonstrated a ‘bias blind spot’—that people tend to perceive themselves as less susceptible to biases than others. A recent replication based on a larger and more diverse sample supported these findings, also showing links between free will agency beliefs and the bias blind spot (Chandrashekar et al. 2021). An interesting direction for future research, would be to examine whether given the self-other asymmetries regarding the bias blind-spot and agency, and given the links between the agency beliefs and the bias blind-spot, that there might be a common mechanism underlying these types of effects.

Our Study 2 also offers an important insight given the comparison of the between-subject and within-subject design. We found support for an effect in a between-subject design but no support for the effect using the within-subject design. This experimental contrast has been commonly used in the judgment and decision-making literature in order to demonstrate that when comparisons are possible people realize the bias and correct it. For example, Hsee (1998) provided several curious demonstrations of the ‘Less Is Better’ effect when people evaluate objects or situations in isolation (separately, in a between-subject design) versus the more rational ‘More Is Better’ effect when people evaluate those in joint evaluations. His findings were recently successfully replicated by Vonasch et al. (2023). Therefore, it would seem that in our current replication, like in Hsee (1998) and Vonasch et al. (2023), the effect seems to appear when evaluations are done in isolation with no reference point for comparison, yet seems to dissolve when making joint evaluations.

While our findings suggest that the perceived predictability asymmetry is more robust in a between-subject design compared to a within-subject design, the reason as to why remains unclear and suggests needed future research. A reasonable explanation would be that in within-subject designs people use the information about self and other to make comparisons and adjustments, which is not directly possible in the between-subject design. To better understand what is driving these differences and whether participants indeed do make comparisons would require additional measures to capture the thought processes during the tasks. Future research may help clarify whether the observed effects are indeed due to differences in evaluative modes or if other underlying mechanisms are at play. Perhaps one way to tease apart the variation in the findings across between-persons versus within-person comparisons is to investigate the normative understanding of free will, in its different descriptions. A valuable avenue for future research could involve investigating whether individuals tend to favor or trust others who exhibit less predictability, and under what circumstances. Likewise, an intriguing line of inquiry would be to examine whether individuals express a preference for or trust those who demonstrate a heightened sense of agency.

In the replication of Study 1a which closely matched the target study, we found strong support for predictions of the original study (*d* = –0.84, 95% CI [–1.46, –0.23]) based on a smaller student sample (*N* = 47). In Study 1b with a larger MTurk sample (*N* = 126) the replication effect size seemed much weaker and just barely detectable signal (*d* = –0.30, 95% CI [–0.66, 0.05]), whereas in Study 2, which had the largest sample, the effect with a between-subject design was again smaller than yet in-line with the original’s (*d* = –0.33, 95% CI [–0.52, –0.14]). As a whole, we consider this a mostly successful replication of the main finding in Pronin and Kugler: Experiment 1 (2010), although the observed effect sizes were smaller. For future studies aiming to replicate and extend these findings further, for example by looking into underlying mechanisms and testing potential moderators, we would recommend recruiting a much larger sample size.

The current investigation, specifically Study 2, also aimed to test the moderating effect of achievement motivation; however, the findings did not support our predictions. We found no evidence of a relationship between achievement motivation and the tendency to view oneself as less predictable than others. Achievement motivation is typically associated with goal-directed behavior and a sense of agency (Lang & Fries 2006), yet we failed to find any indication of an association with unpredictability, as a measure of free will attributions. Future research may explore whether other dimensions of agency are predictive of unpredictability or other aspects of the notion of free will.

## Unpredictability and Free Will: Challenges in Measurement and Interpretation

Pronin and Krugler (2010) argued that unpredictability as it is measured in the original study maps onto indeterminism. The idea was that an individual’s actions are not preordained and cannot be predicted in advance. Our understanding was that there was an implicit assumption that unpredictability as a tenant of free will is due to the potential for variations in one’s own goals, intentions, or behaviors rather than unpredictability depending on unchosen dispositions or uncontrolled contextual influences. Pronin and Krugler (2010) suggested that people who view their own decisions and life events as less predictable compared to those of others seem to perceive having more choice and control over their behavior, seeing themselves as more capable of overcoming both external challenges and internal limitations. However, the study methods left it unclear whether participants rated higher unpredictability as a direct reflection of personal free will or because of other reasons, such as perceiving others’ as being worse than they are at the mere task of prediction. The commonly used survey instruments that aim to capture individuals’ belief in free will do not, at least directly, mention unpredictability (e.g., Nadelhoffer et al. 2014; Paulhus & Carey 2011; Rakos et al. 2008).

Additionally, the valence associated with the questions for which study participants rated unpredictability remains unclear (e.g., attending a specific university, choosing a major, choosing a dating partner). Contrary to Pronin and Kugler’s (2010) findings, previous research suggests that predictability of behavior might be considered desirable when such behaviors conform to social norms (Codol 1975) or when the behaviors in question relate to morality (Turpin et al. 2021). Although the measure of unpredictability appears neutral, participants may have perceived it differently. Future investigations could benefit from including additional measures that account for the valence of the outcomes or behaviors for which unpredictability is evaluated.

## Additional File

The additional file for this article can be found as follows:

10.5334/irsp.946.s1 Supplementary Materials. Tables S1 to S17.

## Footnotes

In our pre-registration, we referenced one-tailed power analyses but did not specify whether we intended to use one-tailed or two-tailed tests. We present the data as is, adhering to our pre-registered criteria to limit analytical flexibility, while acknowledging the challenges posed by p-values just below .05. Given the clear directional hypotheses associated with the replication, reporting one-tailed tests appears justified.

The mixed condition was planned (based on the pre-registration) to mirror the original study’s mixed allocation (i.e., self-other is between-subject, past-future is within-subject). The actual presentation of conditions during the execution of the study made it impossible to conduct a straightforward test (i.e., ANOVA or t-tests) of the original predictions. A total of 437 (participants assigned to mixed-condition) of the 1295 participants in the sample were in the excluded condition. An additional analysis (i.e., a mixed-effect regression analysis) based on the executed mixed-condition is reported in the supplementary materials.

## Peer Review comments

IRSP Open Peer Review: https://osf.io/d47kj.
