# Supplementary material for: Do People Believe They Are Less Predictable Than Others? Three Replications of Pronin and Kugler’s (2010) Experiment 1
Source: Int Rev Soc Psychol. 2024 Dec 6;37:20. doi: 10.5334/irsp.946 (PMC12372692; doi:10.5334/irsp.946)
Supplement: Supplementary Materials. — Tables S1 to S17. [file irsp-37-946-s1.pdf]

Do people believe they are less predictable than others? Three replications of Pronin and Kugler (2010)'s Experiment 1

<u>Supplementary</u>

Overview of Experimental Design in the Target article 2
Experimental design 4
Experimental material 4
Original article's results 5
Effect size calculations of the target article effects 6
Power analyses 7
Gpower protocols 8
Materials and scales related to replication part 9
Study Materials: Study 1a & 1b 9
Study Materials: Study 2 10
Deviations from Preregistration 13
Additional results 14
Study 1a 14
Study 1b 14
Study 2 15
Study 2: Mixed design: Past versus future separately for self and for other 16
Study 2 **Extension: Achievement Motives** 18
Introduction 18
Results 18
Results of Mini-meta analysis 20
Mini-meta-analyses including the original study 20
Mini-meta analyses excluding the original study 23
Bayesian analyses 25
References 30

Author Bios:

Subramanya Prasad Chandrashekar is postdoctoral researcher with the Department of Psychology at the Norwegian University of Science and Technology (NTNU). His research focuses on moral psychology, lay-beliefs, and judgment and decision-making.

Stephanie Permut is an associate fellow with the Andlinger Center for Energy and the Environment at Princeton University.

Hallgeir Sjåstad is a professor at the Norwegian School of Economics, Department of Strategy and Management. His research focuses on judgment and decision-making, and social psychology.

Chi Wing LO, Yong Jun KUEH, Emily Sihui ZHONG, Kai Hin WAN, Kay Yi Kelly CHOY, Man Chung WONG, Stanley Wei Jian HUGH, and Khan TAHIRA were students at the University of Hong Kong in the year 2018.

Bo Ley Cheng was a teaching assistant at the University of Hong Kong psychology department in the year 2018.

Gilad Feldman is an assistant professor with the University of Hong Kong psychology department. His research focuses on judgment decision-making.

Declaration of Conflict of Interest:

The author(s) declared no potential conflicts of interests with respect to the authorship and/or publication of this article.

Financial disclosure/funding:

The author(s) received no financial support for the research and/or authorship of this article.

Acknowledgments or Author Notes:

None.

Authorship declaration

A team comprising of Chi Wing LO, Yong Jun KUEH designed and wrote the pre-registrations for Study 1b. A team comprising of Emily Sihui ZHONG, Kai Hin WAN, Kay Yi Kelly CHOY, Man Chung WONG, Stanley Wei Jian HUGH, and Khan TAHIRA designed and wrote the pre-registrations for Study 1a. Bo Ley CHENG and Gilad Feldman guided the two teams. Gilad conducted the pre-registrations, and ran data collections. The two teams analyzed and wrote initial reports of the findings.

Hallgeir and Gilad designed and wrote the pre-registration for Study 2. Gilad conducted Study 2 data collection. Prasad and Stephanie verified teams' work on Studies 1a and 1b, and analyzed Study 2.

Hallgeir and Stephanie integrated the three projects to a unified manuscript. Gilad reverified the three studies, added analyses, and edited the first draft. Prasad verified and extended the analyses and finalized the manuscript for journal submission, with Gilad's final editing.

Contributor Roles Taxonomy

Role	S.P. Chandrashekar	Hallgeir Sjåstad	Stephanie Permut	Chi Wing LO, Yong Jun KUEH, Emily Sihui ZHONG, Kai Hin WAN, Kay Yi Kelly CHOY, Man Chung WONG, Stanley Wei Jian HUGH, and Khan TAHIRA	Bo Ley CHENG	Gilad Feldman
Conceptualization						X
Pre-registrations		X		X		X
Data curation						X
Formal analysis	X		X	X		X
Funding acquisition						X
Investigation				X		X
Methodology				X		X
Pre-registration peer review / verification				X	X	X
Data analysis peer review / verification	X	X	X	X		X
Project administration					X	X
Resources						X
Software	X			X		
Supervision		X			X	X
Validation	X		X			X
Visualization	X		X			X
Writing-original draft	X	X	X			X
Writing-review and editing	X	X	X			X

Overview of Experimental Design in the Target article

Experimental design

Original study authors ran 2(between) x 2 (within) mixed-design ANOVA comparing self-other difference and past- future difference in the predictability of personal action.

Experimental material

Other/close friend

Please think about one of your roommates (or suitemates) at Princeton. Pick one person only, and indicate his/her initials here: ____. If you do **not** have a roommate or suitemate, choose one you previously had, and indicate his/her initials here: ____.

Looking back…

1. Think about your roommate's decision to attend Princeton. How easy would it have been to predict that he/she would end up going to Princeton? (Not at all predictable = 1; Extremely predictable = 7)
2. Think about a person whom your roommate recently dated (but is not dating any longer). How easy would it have been to predict that things would not last between the two of them? (Not at all predictable = 1; Extremely predictable = 7)
3. Think about your roommate's choice of what to major in. How easy would it have been to predict that he/she would end up choosing that major? (Not at all predictable = 1; Extremely predictable = 7)

Looking forward…

1. Think about what your roommate will end up doing for his/her career. How easy would it be to predict what his/her career will be? (Not at all predictable = 1; Extremely predictable = 7)
2. Think about the person whom your roommate will marry. How easy would it be to predict who this person will be? (Not at all predictable = 1; Extremely predictable = 7)
3. Think about your roommate's decision about where in the country he/she will live during the decade after he/she graduates. How easy would it be to predict where he/she will live? (Not at all predictable = 1; Extremely predictable = 7)

Self

Looking back…

1. Think about your decision to attend Princeton. How easy would it have been to predict that you would end up going to Princeton?
2. Think about a person whom you recently dated (but are not dating any longer). How easy would it have been to predict that things would not last between the two of you?
3. Think about your choice of what to major in. How easy would it have been to predict that you would end up choosing that major?

Looking forward…

1. Think about what you will end up doing for your career. How easy would it be to predict what your career will be?
2. Think about the person whom you will marry. How easy would it be to predict who this person will be?
3. Think about your decision about where in the country you will live during the decade after you graduate. How easy would it be to predict where you will live?

Original article's results

We calculated the effect sizes based on the results of the target study. See **Appendix A** for sample calculation of Cohen's d (95% CI intervals).

Prediction 1: *Participant perceived their own outcome as less predictable a priori than those of a roommate (the main effect of a priori predictability of self and others' past and future decision)*

- Mean = 3.86(self) vs. 4.81(other), (on a 7-point scale ranging from 1 = not at all predictable to 7 = extremely predictable);
- Standard Deviation = Not Reported
- The Statiscal Test: two-way ANOVA: F(1, 48) = 14.46
- The reported degree of freedom = 48
- The reported p-value = .0004.
- Cohen's d = -1.08 [-1.68, -0.47]

Prediction 2: *The simple effect for self-other difference for past outcomes*

- Mean = 4.32(self) vs. 5.16(other), (on a 7-point scale ranging from 1 = not at all predictable to 7 = extremely predictable)
- Standard Deviation = Not Reported
- The Statistical Test: two-way ANOVA: F(1, 48) = 6.40
- The reported degree of freedom = 48
- The reported p-value = .01
- Cohen's d = -0.72 [-1.30, -0.13]

Prediction 3: *The simple effect for self-other difference for future outcomes*

- Mean = 3.40(self) vs. 4.47(other), (on a 7-point scale ranging from 1 = not at all predictable to 7 = extremely predictable)
- Standard Deviation = Not Reported
- The Statistical Test: two-way ANOVA: F(1, 48) = 8.46
- The reported degree of freedom = 48
- The reported p-value = .005
- Cohen's d = -0.82 [-1.41, -0.23]

Prediction 3: *The differences for future vs past outcome predictabilities*

- Mean = 3.93(self) vs. 4.74(other), (on a 7-point scale ranging from 1 = not at all predictable to 7 = extremely predictable)
- Standard Deviation = Not Reported
- The Statistical Test: two-way ANOVA: F(1, 48) = 10.94
- The reported degree of freedom = 48
- The reported p-value = .002
- Cohen's d = -0.94 [-1.53, -0.34]

Effect size calculations of the target article effects

Effect sizes were not reported in the target article. We thus computed these whenever possible (when there was sufficient information).

Table S1. Summary of target findings in Pronin and Kugler (2010) Experiment 1

	df 1	df 2	F	p	Cohen's d with 95% CI	Partial eta-square with 90% CI
(H1) Across both past and future outcomes: Self vs. Roommate	1	48	14.46	.0004	-1.08 [-1.68, -0.47]	0.23 [0.11, 1.00]
(H2a) Across both past outcomes: Self vs. Roommate	1	48	6.40	.010	-0.72 [-1.30, -0.13]	0.12 [0.03, 1.00]
(H2b) Across both future outcomes: Self vs. Roommate	1	48	8.46	.005	-0.82 [-1.41, -0.23]	0.15 [0.05, 1.00]
(H3) Future vs. Past	1	48	10.94	.002	-0.94 [-1.53, -0.34]	0.19 [0.07, 1.00]

Power analyses

Steps for power analysis (Pronin & Kugler, 2010):

These are the results reported in the target study:

> *Participants perceived their own outcomes as less predictable a priori than those of a roommate (M = 3.86 vs. 4.81, on a 7-point scale ranging from 1 = not at all predictable to 7 = extremely predictable); F (1, 48) = 14.46, p = .0004. This self–other difference was significant for both past outcomes (M = 4.32 vs. 5.16), F (1, 48) = 6.40, P = 0.01 and future outcomes (M = 3.40 vs. 4.47), F (1, 48) = 8.46, p = .005. Consistent with previous theorizing (1), there also was a main effect for time, whereby people perceived the future as less predictable than the past (M = 3.93 vs. 4.74); F (1, 48) = 10.94, p = .002. There was no interaction between past/future and self/other (F < 1).*

Here is a summarised form of the statistics of the results:

- General: Self vs other (M = 3.86 vs 4.81, SD not provided), F (1, 48) = 14.46, p = .0004
- Past outcomes: Self vs other (M = 4.32 vs 5.16, SD not provided), F (1, 48) = 6.40, p = .01
- Future outcomes: Self vs Other (M = 3.40 vs 4.47, SD not provided), F (1, 48) = 8.46, p = .005
- Future vs Past (M = 3.93 vs 4.74, SD not provided), F (1, 48) = 10.94, p = .002

Standard deviations were not provided so we took the F statistic and the overall number of subjects to

We provided reproducible Rmarkdown code and output in files PK2010repext-RNR-effect-calc-power-analysis.Rmd/html in the OSF folder, which mirror and verify the GPower (Faul et al., 2007) analyses below.

Gpower protocols

<table>
<tr><td>Power analysis output for Study 1a and Study 1b</td><td>G*Power output
Analysis: A priori: Compute required sample size
Input: Tail(s) = Two
Effect size d = 1.1
α err prob = 0.05
Power (1-β err prob) = 0.95
Allocation ratio N2/N1 = 1
Output: Noncentrality parameter δ = 3.7302815
Critical t = 2.0153676
Df = 44
Sample size group 1 = 23
Sample size group 2 = 23
Total sample size = 46
Actual power = 0.9542682

Considering the effect size of the general main effect which is the most crucial to the experiment, d = 1.1. Hence, a sample size of 23 participants in each condition is required for two-tail power .95 alpha .05. Therefore, a sample of 46 participants is required.</td></tr>
<tr><td>Power analysis output for Study 2</td><td>Cohen's d = 0.32 based on the results of the Study 1b
G*Power output
t tests - Means: Difference between two independent means (two groups)
Analysis: A priori: Compute required sample size
Input: Tail(s) = One
Effect size d = 0.32
α err prob = 0.05
Power (1-β err prob) = 0.95
Allocation ratio N2/N1 = 1
Output: Noncentrality parameter δ = 3.3023628
Critical t = 1.6484553
Df = 424
Sample size group 1 = 213
Sample size group 2 = 213
Total sample size = 426
Actual power = 0.9507565</td></tr>
</table>

Materials and scales related to replication part

Study Materials: Study 1a & 1b

OSF Link to the Qualtrics survey file (in word doc format):

Study 1a: https://osf.io/qmhy

Study 1b: https://osf.io/n6gsy

Introduction to evaluations of others

First, please think about your closest friend or your roommate at your university.
Pick **one** person only and indicate his/her first name initials here (do NOT write full name or any identifying details, to ensure full anonymity):

Past events in others' lives

Read the following statements about certain past events. Think about how predictable those events were in your friend/roommate's life and rate them on a scale from 1 (not at all predictable) to 7 (extremely predictable).

1. Think about your friend/roommate's decision to attend your university. How easy would it have been to predict that he/she would end up going to this university? (1= Not at all predictable; 7 = Extremely predictable)
2. Think about a person whom your friend/roommate recently dated (but is not dating any longer). How easy would it have been to predict that things would not last between the two of them? (1= Not at all predictable; 7 = Extremely predictable)
3. Think about your friend/roommate's choice of what to major in. How easy would it have been to predict that he/she would end up choosing that major? (1= Not at all predictable; 7 = Extremely predictable)

Future events in others' lives

Read the following statements about certain future events. Think about how predictable those events are **in your friend/roommate's life** and rate them on a scale from 1 (not at all predictable) to 7 (extremely predictable).

1. Think about what your friend/roommate will end up doing for his/her career. How easy would it be to predict what his/her career would be? (1= Not at all predictable; 7 = Extremely predictable)
2. Think about the person whom your friend/roommate will marry. How easy would it be to predict who this person would be? (1= Not at all predictable; 7 = Extremely predictable)
3. Think about your friend/roommate's decision about where he/she will live during the decade after he/she graduates. How easy would it be to predict where he/she would live? (1= Not at all predictable; 7 = Extremely predictable)

Introduction to evaluations of self

In this section you're asked to evaluate **events in your own life**.

Read the following statements about **past events in your life**. Think about how predictable those events are and rate them on a scale from 1 (not at all predictable) to 7 (extremely predictable).

1. Think about your decision to attend your current university. How easy would it have been to predict that you would end up going to this university? (1= Not at all predictable; 7 = Extremely predictable)
2. Think about a person whom you recently dated (but are not dating any longer). How easy would it have been to predict that things would not last between the two of you? (1= Not at all predictable; 7 = Extremely predictable)
3. Think about your choice of what to major in. How easy would it have been to predict that you would end up choosing that major? (1= Not at all predictable; 7 = Extremely predictable)

Future events in your own life

Read the following statements about **future events in your life**. Think about how predictable those events are and rate them on a scale from 1 (not at all predictable) to 7 (extremely predictable).

1. Think about what you will end up doing for your career. How easy would it be to predict what your career would be? (1= Not at all predictable; 7 = Extremely predictable)
2. Think about the person whom you will marry. How easy would it be to predict who this person would be? (1= Not at all predictable; 7 = Extremely predictable)
3. Think about your decision about where you will live during the decade after you graduate. How easy would it be to predict where you would live? (1= Not at all predictable; 7 = Extremely predictable)

Study Materials: Study 2

OSF Link to the Qualtrics survey file (in word doc format):

Study 2: https://osf.io/vx4pa

Introduction: Students only
This experiment is only for current American undergraduate students.

This experiment has 6 questions presented in one page asking for you evaluations of behaviors. There are no right or wrong answers, answer to the best of your understanding.

Are you currently an American undergraduate student?

- Yes, I am currently an undergraduate student (1)
- No, I am not an undergraduate student (2)

What year of your undergraduate studies are you currently at?

- First year (1)
- Second year (2)
- Third year (3)
- Forth year+ (4)
- Not currently enrolled (5)

Achievement motivations

The following questions are meant to understand your motivations for achievement.
Please indicate your agreement with each of the following statements: (*1= Strongly disagree; 4 = Strongly agree*)

1. I like situations, in which I can find out how capable I am.
2. When I am confronted with a problem, which I can possibly solve, I am enticed to start working onit immediately.
3. I enjoy situations, in which I can make use of my abilities.
4. I am appealed by situations allowing me to test my abilities.
5. I am attracted by tasks, in which I can test my abilities.
6. I am afraid of failing in somewhat difficult situations, when a lot depends on me.
7. I feel uneasy to do something if I am not sure of succeeding.
8. Even if nobody would notice my failure, I'm afraid of tasks, which I'm not able to solve.
9. Even if nobody is watching, I feel quite anxious in new situations.
10. If I do not understand a problem immediately I start feeling anxious.

Introduction to evaluations of others

First, please think about your closest friend or your roommate at your university.
Pick **one** person only and indicate his/her first name initials here (do NOT write full name or any identifying details, to ensure full anonymity):

Past events in others' lives

Read the following statements about certain past events. Think about how predictable those events were in your friend/roommate's life and rate them on a scale from 1 (not at all predictable) to 7 (extremely predictable).

4. Think about your friend/roommate's decision to attend your university. How easy would it have been to predict that he/she would end up going to this university? (1= Not at all predictable; 7 = Extremely predictable)
5. Think about a person whom your friend/roommate recently dated (but is not dating any longer). How easy would it have been to predict that things would not last between the two of them? (1= Not at all predictable; 7 = Extremely predictable)
6. Think about your friend/roommate's choice of what to major in. How easy would it have been to predict that he/she would end up choosing that major? (1= Not at all predictable; 7 = Extremely predictable)

Future events in others' lives

Read the following statements about certain future events. Think about how predictable those events are **in your friend/roommate's life** and rate them on a scale from 1 (not at all predictable) to 7 (extremely predictable).

4. Think about what your friend/roommate will end up doing for his/her career. How easy would it be to predict what his/her career would be? (1= Not at all predictable; 7 = Extremely predictable)
5. Think about the person whom your friend/roommate will marry. How easy would it be to predict who this person would be? (1= Not at all predictable; 7 = Extremely predictable)
6. Think about your friend/roommate's decision about where he/she will live during the decade after he/she graduates. How easy would it be to predict where he/she would live? (1= Not at all predictable; 7 = Extremely predictable)

Introduction to evaluations of self

In this section you're asked to evaluate **events in your own life**.

Read the following statements about **past events in your life**. Think about how predictable those events are and rate them on a scale from 1 (not at all predictable) to 7 (extremely predictable).

4. Think about your decision to attend your current university. How easy would it have been to predict that you would end up going to this university? (1= Not at all predictable; 7 = Extremely predictable)
5. Think about a person whom you recently dated (but are not dating any longer). How easy would it have been to predict that things would not last between the two of you? (1= Not at all predictable; 7 = Extremely predictable)
6. Think about your choice of what to major in. How easy would it have been to predict that you would end up choosing that major? (1= Not at all predictable; 7 = Extremely predictable)

Future events in your own life

Read the following statements about **future events in your life**. Think about how predictable those events are and rate them on a scale from 1 (not at all predictable) to 7 (extremely predictable).

4. Think about what you will end up doing for your career. How easy would it be to predict what your career would be? (1= Not at all predictable; 7 = Extremely predictable)
5. Think about the person whom you will marry. How easy would it be to predict who this person would be? (1= Not at all predictable; 7 = Extremely predictable)
6. Think about your decision about where you will live during the decade after you graduate. How easy would it be to predict where you would live? (1= Not at all predictable; 7 = Extremely predictable)

Deviations from Preregistration

Table S2. *Pre-registration plan versus final report*

**Components in preregistration**	**Location of preregistered decision/plan**	**Deviations**	**Description of deviation**	**Rationale for deviation**	**Impact of deviation on results**	**Date/time of decision for deviation + stage**
Study design	Study 1a: https://osf.io/j5ms4<br><br>Study 1b: https://osf.io/xwhu3 https://osf.io/tfwrg<br><br>Study 2: https://osf.io/7ucjv	no	/	/	/	/
Measured variables		no	/	/	/	/
Exclusion criteria		no	/	/	/	/
IV		no	/	/	/	/
DV		no	/	/	/	/
Data analysis		Yes	We did not deviate in our analysis yet report the effect using a partial eta-squared effect size measure. Power analysis was based on Cohen's effect size measure.	partial eta-squared is more apt effect size measure for an ANOVA analysis	none	June 2023

P-values alpha

We note that we did not pre-register whether our alpha is for one-tail or two-tail, with the pre-registration for Study 1a noting power analysis for two-tail and the pre-registration for Study 1b noting one-tail. In the reported results below, we refer to one-tail tests, given that the hypotheses are well-stated and these were replications.

Additional results

Study 1a

Table S3
Study 1a: Results of mixed ANOVA

**Effect**	***df***	**MSE**	***F***	***p***	η_p^2 **(90% CI)**
Self vs. Other	1, 45	1.42	8.33	.006	0.16 [0.05, 1.00]
Future vs. Past	1, 45	1.32	16.26	<.001	0.27 [0.13, 1.00]
(Self vs. Other) x (Future vs. Past)	1, 45	1.32	1.35	.251	0.03 [0.00, 1.00]

Table S4
Study 1a: Results of post hoc comparisons based on mixed ANOVA

**Contrast**	**estimate**	**SE**	***df***	***t***	***p***	**Cohen's *d*, 95% CI**
Future						
Self - Other	-0.99	0.36	45	-2.72	.0092	-0.79 [-1.40, -0.18]
Past						
Self - Other	-0.43	0.32	45	-1.36	.1817	-0.40 [-0.99, -0.20]

Study 1b

Table S5
Study 1b: Results of mixed ANOVA

**Effect**	***df***	**MSE**	***F***	***p***	η_p^2 **with (90% CI)**
Self vs. Other	1, 124	2.25	2.87	.093	0.02 [0.00, 1.00]
Past v. Future	1, 124	0.74	26.22	<.001	0.17 [0.10, 1.00]
(Self vs. Other) x (Past vs. Future)	1, 124	0.74	0.42	.516	0.00 [0.00, 1.00]

Table S6
Study 1b: Results of post hoc comparisons based on mixed ANOVA

**Contrast**	**estimate**	**SE**	***df***	***t***	***p***	**Cohen's *d*, 95% CI**
Future						
Self - Other	-0.39	0.24	124	-1.626	.106	-0.29 [-0.64, -0.06]
Past						
Self - Other	-0.25	0.19	124	-1.29	.198	-0.23 [-0.58, -0.12]

Study 2

Table S7

Study 2: Results of ANOVA based on responses from between-subjects design

Effect	*df*	MSE	*F*	*p*	η_p^2 with (95% CI)
Self vs. Other	1, 420	0.82	12.59	<.001	0.03 [0.01, 1.00]
Past v. Future	1, 420	0.82	37.74	<.001	0.08 [0.05, 1.00]
(Self vs. Other) x (Past vs. Future)	1, 420	0.82	2.40	.122	0.00 [0.00, 1.00]

Table S8

Study 2: Results of post hoc comparisons based on ANOVA from between-subjects design

Contrast	estimate	SE	*df*	*t*	*p*	Cohen's d, 95% CI
Future						
Self - Other	-0.45	0.12	420	-3.59	<.001	-0.46 [-0.74, -0.18]
Past						
Self - Other	-0.18	0.12	420	-1.42	.156	-0.21 [-0.48, -0.06]

Table S9

Study 2: Results of ANOVA based on responses from within-subjects design

Effect	*df*	MSE	*F*	*p*	η_p^2 with (95% CI)
Self vs. Other	1, 433	0.66	0.00	.988	0.00 [0.00, 1.00]
Past v. Future	1, 433	0.68	105.23	<.001	0.20 [0.15, 1.00]
(Self vs. Other) x (Past vs. Future)	1, 433	0.47	1.43	.233	0.00 [0.00, 1.00]

Table S10

Study 2: Results of post hoc comparisons based on ANOVA from within-subjects design

Contrast	estimate	SE	*df*	*t*	*p*	Cohen's d, 95% CI
Future						
Self - Other	0.04	0.04	433	0.82	.412	0.05 [-0.09, 0.18]
Past						
Self - Other	-0.04	0.05	433	-0.72	.469	-0.04 [-0.17, -0.09]

Study 2: Mixed design: Past versus future separately for self and for other

We also added a condition with a design in which participants rated the predictability of past or future events across each of the self and other's reference point. The participants in the mixed condition were assigned to one of the four scenario combinations: 1) Self-past and Other-past; 2) Self-past and Other-future; 3) Self-future and Other-past; 4) Self-future and Other-future. The order of the presentations within the combinations was randomized. We summarized descriptive in Table 5 and Figure 5. The means were very similar to that of the within-subject condition, with no differences between self and other.

Table S11.
Study 2 mix design: Predictability descriptives

Target [Between]	Self		Other	
Time [within]	*M*	*SD*	*M*	*SD*
Past	3.78	0.75	3.68	0.77
Future	3.22	0.94	3.22	0.95

Note. *N* = 437.

Figure S4.
Study 2 mix design: Self-other predictability separately for past and future choices

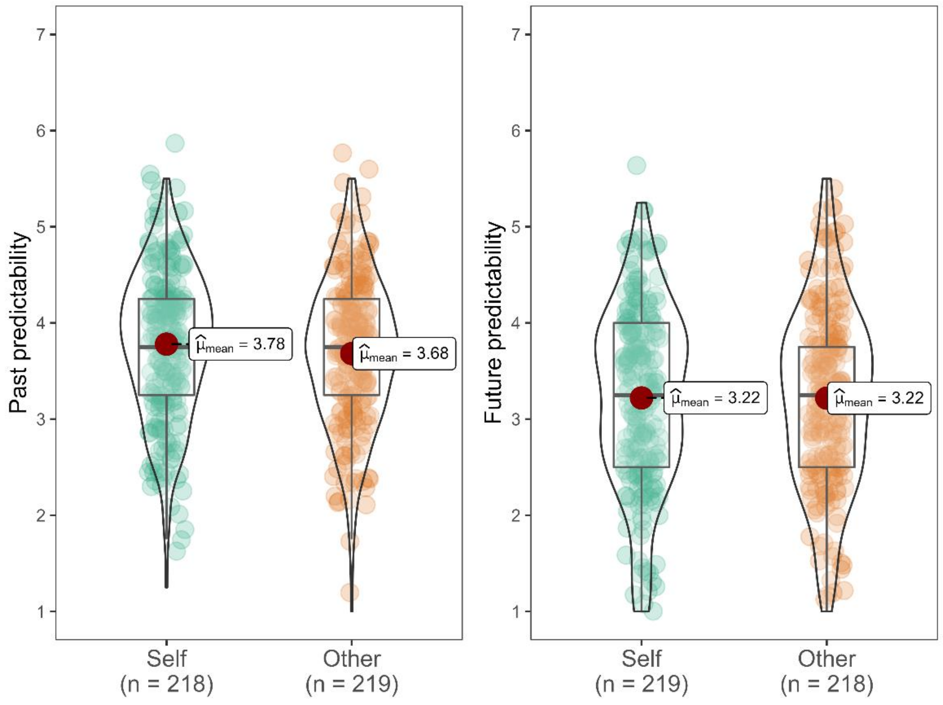

Given the nature of mixed experimental design, we conducted a mixed effects regression analysis. We regressed predictability ratings on target frame (self vs. other), time factor (past vs future), and interaction between reference frame and time factor as fixed effects predictors, and participants' ID as the random intercept. We constructed the linear mixed model (using the *lme4* package in R; Bates et al., 2015) in R. The *p* values were calculated using the Satterthwaite approximation for the mixed-effects regressions (Kuznetsova et al., 2017). The results of the analysis are presented in the Table 12. There was no support for target frame as a predictor. However, we found support for the view that future events (M= 3.22; SD= 0.94) are rated less predictable than the past events (M= 3.73; SD= 0.76; estimate = -0.57, 95% CI [-0.73 – -0.41], *p* <.001).

Table S12

Study 2: Results of mixed-effect regression analysis

	**DV: Predictability**				
Predictors	*b*	*se*	*CI*	*t-value*	*p*
(Intercept)	3.78	0.06	3.67 – 3.90	65.44	**<0.001**
Target frame (Other vs. Self)	-0.10	0.08	-0.25 – 0.06	-1.22	.222
Time (Future vs. Past)	-0.57	0.08	-0.73 – -0.41	-6.98	**<0.001**
Target frame × Time	0.09	0.12	-0.13 – 0.32	0.81	.420
**Random Effects**					
σ^2				0.65	
τ_{00} ParticipantID				0.08	
ICC				0.11	
N ParticipantID				437	
Observations				874	
Marginal R^2 / Conditional R^2				0.086 / 0.191	

Study 2 Extension: Achievement Motives

Introduction

Our Study 2 also included a second extension in which we measured individual differences in achievement motivation (Lang & Fries, 2006) as a potential trait moderator. Since a high need for achievement is positively associated with work ethic and performance, it could well be the case that the ego-centric effect in predictability judgments is more pronounced for participants with high rather than low achievement motivation. Such a result would provide initial correlational evidence that the effect is associated with motivated beliefs.

Table S13
Summary of extension hypotheses

Study	Hypothesis
Hypothesis 4	People with high (vs. low) achievement motivation will have a stronger tendency to think that they have more free will than others (i.e., less predictable)
Hypothesis 5	People with high (vs. low) achievement motivation will show a greater future vs. past discrepancy.

Results

Our Study 2 involved experimental designs in which the Target (self vs. other) and Time (past vs future) varied as a between or within subject factors. Although, we presented the results independently within each designed, they are less helpful in summarizing the results across the sample. Therefore, we conducted a linear mixed-effects analysis to draw summary conclusions. We pooled the responses across experimental conditions to conduct a linear mixed-effects analysis. Linear mixed-effects models (LMEMs) have several advantages. More importantly, mixed effect analysis is appropriate for our extension hypothesis that proposed the moderating role of the achievement motive measured at the individual level (Lang & Fries, 2006). The results of the analysis are presented in Table 10.

We found no support for differences between self and other. The results of interaction as test of Hypothesis 4 and 5. The results suggest no support the prediction (H4) that people with high (vs. low) achievement motivation will have a stronger tendency to think view outcomes are less predictable than for others (estimate = -0.10, 95% [-0.28, 0.08]), p = .291). Similarly, we found no support for the prediction (H5) that people with high (vs. low) achievement motivation will show a greater future vs. past discrepancy (estimate = 0.01, 95% [-0.17, 0.20]), p = .896). Besides, results held support for the view that future events as more predictable than participants who rated future events.

Table S14

Study 2: Results of mixed effect regression analysis

	Predictability				
Predictors	*b*	*se*	*CI*	*t-value*	*p*
(Intercept)	2.8	0.20	2.96 – 3.72	14.15	**<0.001**
Perspective (Other vs. Self)	0.71	0.25	-0.26 – 0.72	2.81	.365
Time (Past vs. Future)	0.54	0.25	-1.04 – -0.04	2.12	**.034**
Achievement Motivation Scale (AMS)	0.15	0.07	-0.01 – 0.28	1.99	.067
Perspective × Time	-0.48	0.36	-0.23 – 1.19	-1.33	.183
Perspective × AMS	-0.24	0.09	-0.28 – 0.08	-2.57	.291
Time × AMS	-0.01	0.09	-0.17 – 0.20	-0.13	.896
Perspective × Time × AMS	0.14	0.13	-0.40 – 0.12	1.06	.290
**Random Effects**					
σ^2	0.61				
$\tau_{00\ \text{ParticipantID}}$	0.22				
ICC	0.26				
$N_{\text{ParticipantID}}$	1295				
Observations	3034				
Marginal R^2 / Conditional R^2	0.062 / 0.308				

Results of Mini-meta analysis

<u>Mini-meta-analyses including the original study</u>

We employed a fixed-effect model with the REML estimation method for all the meta-analysis results reported here.

Forest plots of the mini meta-analyses. CI = confidence interval. ES = effect size (Cohen's d).

Figure S5

Testing H1 (People would view their own past and future decision as less predictable a priori than those of a roommate.); fixed-effect meta analysis model with the REML estimation

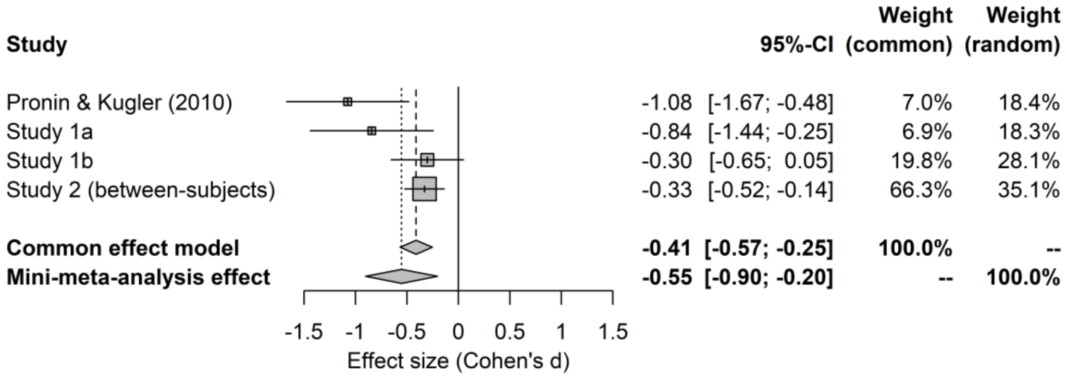

Figure S6

Testing H2a (People will consider their own past decisions as less predictable than the ones of others.); fixed-effect meta analysis model with the REML estimation

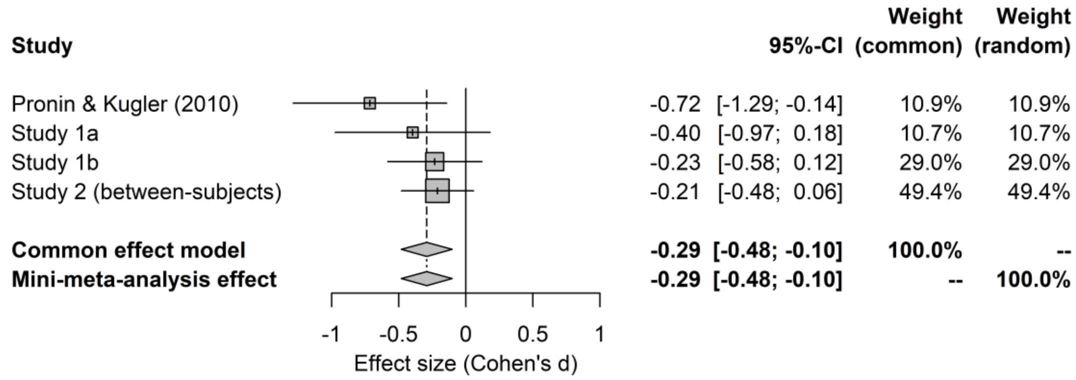
Study
95%-CI
Weight (common)
Weight (random)
Pronin & Kugler (2010) -0.72 [-1.29; -0.14] 10.9% 10.9%
Study 1a -0.40 [-0.97; 0.18] 10.7% 10.7%
Study 1b -0.23 [-0.58; 0.12] 29.0% 29.0%
Study 2 (between-subjects) -0.21 [-0.48; 0.06] 49.4% 49.4%
Common effect model -0.29 [-0.48; -0.10] 100.0% --
Mini-meta-analysis effect -0.29 [-0.48; -0.10] -- 100.0%
-1
-0.5
0
0.5
1
Effect size (Cohen's d)

Figure S7

Testing H2b (People will consider their own future decisions as less predictable than the ones of others.) ; fixed-effect meta analysis model with the REML estimation

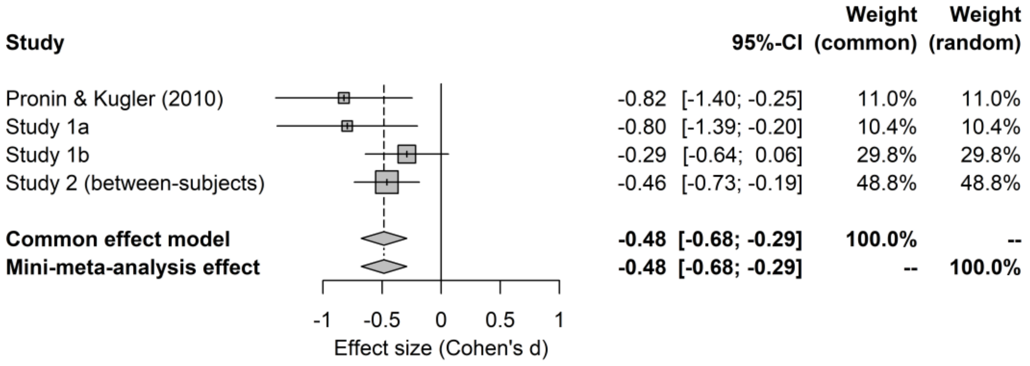

Figure S8. Testing H3 (People would view future decisions as less predictable than past decisions.) ; fixed-effect meta analysis model with the REML estimation

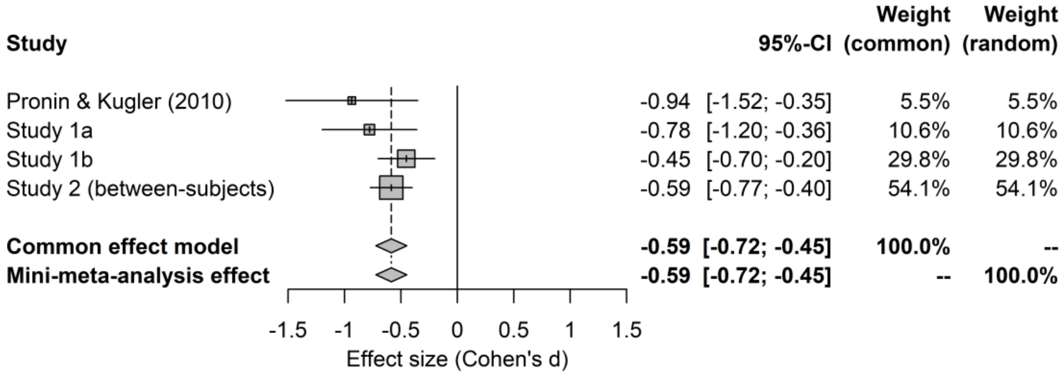

<u>Mini-meta analyses excluding the original study</u>

Note: Forest plots of the mini meta-analyses. CI = confidence interval. ES = effect size (Cohen's d).

Figure S9

Testing H1 (People view their own past and future decision as less predictable a priori than those of a roommate.) ; fixed-effect meta analysis model with the REML estimation

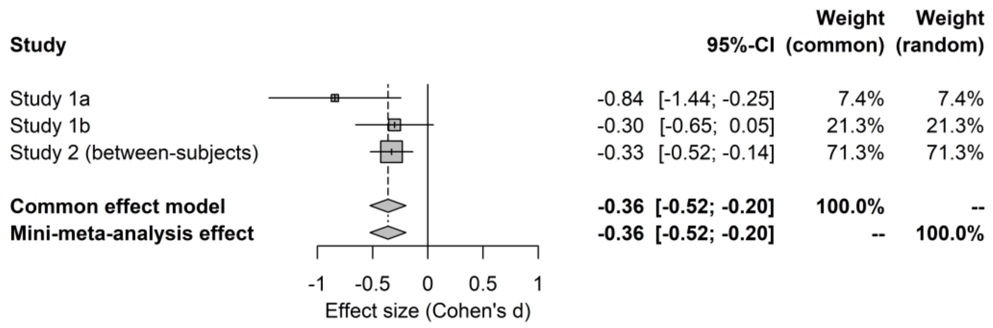

Figure S10

Testing H2a (People consider their own past decisions as less predictable than the ones of others.) ; fixed-effect meta analysis model with the REML estimation

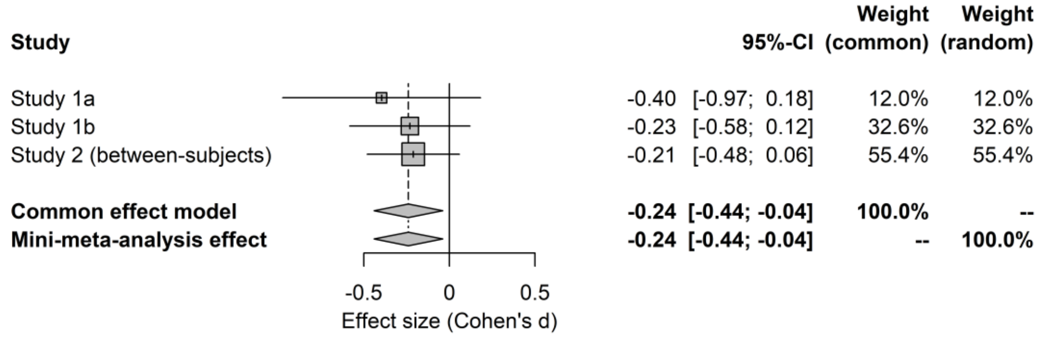

Figure S11

Testing H2b (People consider their own future decisions as less predictable than the ones of others.) ; fixed-effect meta analysis model with the REML estimation

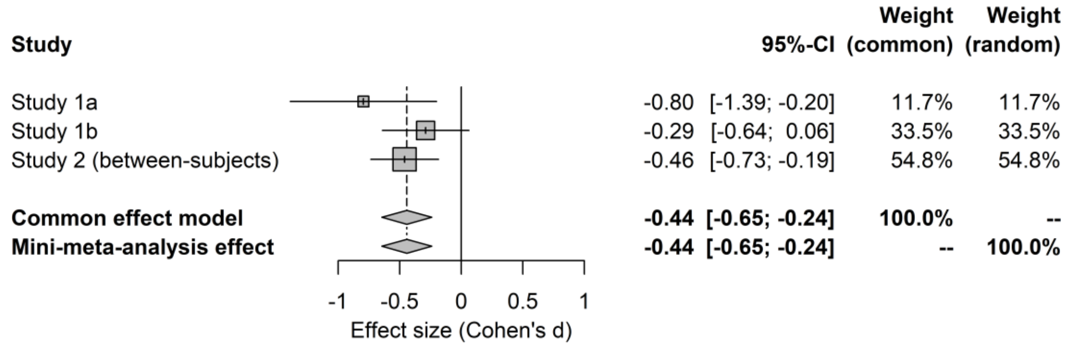

Figure S12

Testing H3 (People view future decisions as less predictable than past decisions.) ; fixed-effect meta analysis model with the REML estimation

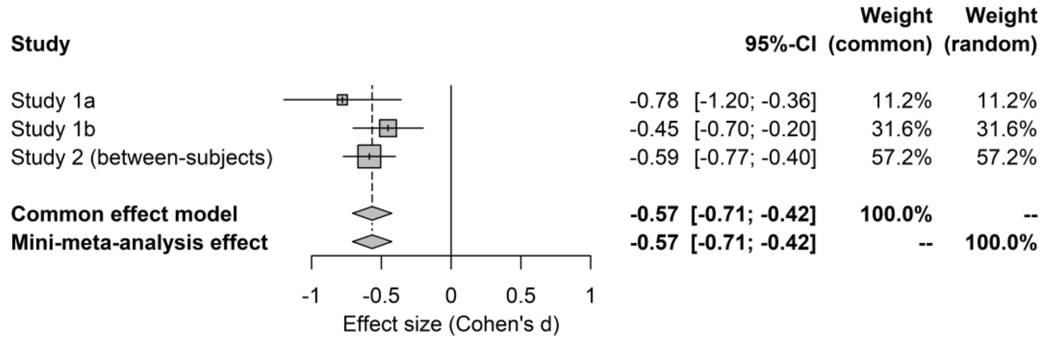

Bayesian analyses

Table S15

Summary of Bayesian analysis results

Study	Analysis	Bayes factor (BF10) with Cauchy prior (0.707)
1a	Past	1.08
	Future	10.34
	Averaged	14.53
1b	Past	0.72
	Future	1.18
	Averaged	1.31
2 Between	Past	0.46
	Future	25.34
	Averaged	25.88

Note. The error % across all the Bayesian analyses was < .000

Moved from the main manuscript

At the request of the editors, due to word count limitations set by the publisher.

Table S15

Comparison of the sample in the original study and in the replications

	Pronin and Kugler (2010), Exp. 1	Replication Study 1a	Replication Study 1b	Replication Study 2
Sample size	50	47	126	858 (of 1295 with excluded condition)
Geographic origin	US American	Hong Kong, SAR	US American	US American
Population	Undergraduates	Undergraduates	MTurk/CloudResearch participants	Prolific participants
Gender	Not reported	21 females	63 females	770 females
Median age	Not reported	20	24	20
Average age	Not reported	20.21	25.83	20.40
Standard deviation age	Not reported	1.02	6.46	1.48
Age range	Not reported	3	36	26
Medium (location)	Not reported	Computer (online)	Computer (online)	Computer (online)
Compensation	Yes, received candy for completing the survey	Yes	Yes	Yes
Year	Probably 2009 or 2010	2018	2018	2019

Table S16

Evaluation of replication closeness (using criteria by LeBel et al., 2018)

**Design facet**	**Replication**	**Details**
Effect/hypothesis	Same	
IV construct	Same	
DV construct	Same	
IV operationalization	Same	
DV operationalization	Same	
Population (e.g., age)	Different	The original study was conducted with a sample of US undergraduates, whereas the replication effort involved a sample of undergraduate students from Hong Kong and US participants from MTurk/CloudResearch and Prolific.
IV stimuli	Some differences for Study 2; Same for Studies 1a and 1b	There are minor set of variations in experimental condition allocations.
DV stimuli	Same	
Procedural details	Similar	
Physical settings	Different	Online data collection in our replication vs. filling a questionnaire<br>in the target article
Contextual variables	Different	The original authors conducted their studies before 2010, whereas we conducted the replications in the years 2018-19.
Replication classification:	Studies 1a, 1b: Very close replication; Study 2: Close replication	

Table S17

Summary of original findings in Pronin and Kugler (2010) Experiment 1

	$F(1, 48)$	p	Effect size Cohen’s d with 95% CI
(H1) Across both past and future outcomes: self vs. other	14.46	.0004	-1.08[-1.67,-0.48]
(H2a) Across past outcomes: self vs. other	6.40	.010	-0.76[-1.29,-0.14]
(H2b) Across future outcomes: self vs. other	8.46	.005	-0.82[-1.40,-0.25]
(H3) Future vs. past	10.94	.002	-0.94[-1.52,-0.35]

Limitations section

Observations, limitations, and future directions

Cultural differences may play a role in how individuals perceive predictability. The original study by Pronin and Kugler (2010) was conducted with U.S. American undergraduates, a demographic that may have distinct cultural attitudes towards individual agency and predictability. Our replication in Hong Kong in Study 1a suggests that the findings extend to a population of a different culture outside the U.S., yet our replications in the U.S. in Studies 1b and 2 with weaker effects suggest that the strength of the effect may vary across samples and contexts. Research on free will and determinism suggested that individuals' beliefs about these concepts may depend on a host of factors, from cultural factors to personal experiences. Future research may continue to explore these dimensions, to continue and test to ensure that findings are not only replicable in the same context but also generalizable across different cultural contexts and methodological approaches, and with meta-analyses that test for moderators to explain differences in effect sizes.

Our replication studies differed from the original study in one key aspect. The original research, conducted in the 2010s, involved U.S. American undergraduate students as participants. In contrast, we administered the same materials, except for Study 1, through online surveys to a more diverse sample population in the USA. Participants recruited on MTurk through CloudResearch were older than the specialized sample used in the original study, for whom the questions may have been less relevant, as the scenario descriptions were most pertinent to undergraduate students. However, we do not see this as a significant issue, since the results from the older and more diverse pool of U.S. American participants still shows support for the original findings. Our replication efforts contribute to the test of the generalizability of the original findings.

References

Pronin, E., & Kugler, M. B. (2010). People believe they have more free will than others. *Proceedings of the National Academy of Sciences*, *107*(52), 22469-22474.

LeBel, E. P., McCarthy, R. J., Earp, B. D., Elson, M., & Vanpaemel, W. (2018). A unified framework to quantify the credibility of scientific findings. *Advances in Methods and Practices in Psychological Science*, *1*, 389-402.

LeBel, E. P., Vanpaemel, W., Cheung, I., & Campbell, L. (2019). A brief guide to evaluate replications. *Meta-Psychology*, 3.
